# *In Vivo* Mitochondrial Function in HIV-Infected Persons Treated with Contemporary Anti-Retroviral Therapy: A Magnetic Resonance Spectroscopy Study

**DOI:** 10.1371/journal.pone.0084678

**Published:** 2014-01-07

**Authors:** Brendan A. I. Payne, Kieren G. Hollingsworth, Joanne Baxter, Edmund Wilkins, Vincent Lee, D. Ashley Price, Michael Trenell, Patrick F. Chinnery

**Affiliations:** 1 Institute of Genetic Medicine, Newcastle University, Newcastle-upon-Tyne, United Kingdom; 2 Department of Infection and Tropical Medicine, Royal Victoria Infirmary, Newcastle-upon-Tyne, United Kingdom; 3 Newcastle Magnetic Resonance Centre, Newcastle University, Newcastle-upon-Tyne, United Kingdom; 4 Department of Infectious Diseases, North Manchester General Hospital, Manchester, United Kingdom; 5 Manchester Centre for Sexual Health, Manchester Royal Infirmary, Manchester, United Kingdom; 6 Institute of Cellular Medicine, Newcastle University, Newcastle-upon-Tyne, United Kingdom; Institute of Infectious Diseases and Molecular Medicine, South Africa

## Abstract

Modern anti-retroviral therapy is highly effective at suppressing viral replication and restoring immune function in HIV-infected persons. However, such individuals show reduced physiological performance and increased frailty compared with age-matched uninfected persons. Contemporary anti-retroviral therapy is thought to be largely free from neuromuscular complications, whereas several anti-retroviral drugs previously in common usage have been associated with mitochondrial toxicity. It has recently been established that patients with prior exposure to such drugs exhibit irreversible cellular and molecular mitochondrial defects. However the functional significance of such damage remains unknown. Here we use phosphorus magnetic resonance spectroscopy (^31^P-MRS) to measure *in vivo* muscle mitochondrial oxidative function, in patients treated with contemporary anti-retroviral therapy, and compare with biopsy findings (cytochrome c oxidase (COX) histochemistry). We show that dynamic oxidative function (post-exertional ATP (adenosine triphosphate) resynthesis) was largely maintained in the face of mild to moderate COX defects (affecting up to ∼10% of fibers): τ_½_ ADP (half-life of adenosine diphosphate clearance), HIV-infected 22.1±9.9 s, HIV-uninfected 18.8±4.4 s, p = 0.09. In contrast, HIV-infected patients had a significant derangement of resting state ATP metabolism compared with controls: ADP/ATP ratio, HIV-infected 1.24±0.08×10^−3^, HIV-uninfected 1.16±0.05×10^−3^, p = 0.001. These observations are broadly reassuring in that they suggest that *in vivo* mitochondrial function in patients on contemporary anti-retroviral therapy is largely maintained at the whole organ level, despite histochemical (COX) defects within individual cells. Basal energy requirements may nevertheless be increased.

## Introduction

Combination anti-retroviral therapy (cART) has transformed the prognosis for HIV-infected persons since the late 1990s. However, patients are at risk of mitochondrial toxicity, thought to be mediated very largely through exposure to certain nucleoside analog reverse transcriptase inhibitor (NRTI) anti-retrovirals. NRTIs were the first class of licensed anti-retroviral drug, and several of the older members of this class, zidovudine, stavudine, zalcitabine and didanosine, are known to inhibit the sole mitochondrial DNA (mtDNA) polymerase, pol γ, resulting in chain termination during mtDNA replication. During therapy, the molecular consequence of this inhibition is reduction in cellular mtDNA content (mtDNA depletion). A wealth of previous studies has demonstrated this phenomenon both *in vitro,* and in a variety of tissues *in vivo* [1]–[4]. These older NRTIs are no longer in common usage in industrialized countries owing to concerns over their toxicity profiles, although zidovudine and stavudine have been very extensively used in anti-retroviral therapy ‘roll-out’ programs in developing countries in recent years. Currently used NRTIs, such as tenofovir (a nucleo*tide* RTI) and abacavir, have been shown to be essentially free from pol γ inhibition *in vitro* and to cause no significant mtDNA depletion *in vivo* [5], [6]. If a patient’s therapy is switched away from a pol γ inhibiting NRTI, the impairment of mtDNA replication is removed and mtDNA content returns to normal [7]. Therefore, although most patients are no longer exposed to pol γ inhibiting NRTIs, a significant cohort of long-term patients will have extensive *prior* exposure to such drugs. Although such patients do not have persistent mtDNA depletion, it has recently been established that they may have persistent histochemical mitochondrial defects evidenced by an increased proportion of COX (cytochrome *c* oxidase) deficient skeletal muscle fibers. These COX deficient fibers contain high levels of individual somatic (acquired) mtDNA mutations (principally large-scale deletion mutations) [8]. The relevance of this persistent cellular and molecular damage on mitochondrial function remains unknown. It is therefore unclear to what extent mitochondria may be functionally impaired in HIV-infected patients treated with contemporary cART.

Phosphorus magnetic resonance spectroscopy (^31^P-MRS) allows the dynamic measurement of *in vivo* skeletal muscle oxidative function through assessment of ATP (adenosine triphosphate) metabolites as well as acid handling. ^31^P-MRS has previously been employed in the longitudinal study of subjects with inherited mitochondrial disorders, both primary mtDNA defects, and secondary mtDNA defects consequent on nuclear gene disorders of mtDNA maintenance [9]–[11]. Limited data also suggests that ^31^P-MRS abnormalities in skeletal muscle may be demonstrated in the setting of acute exposure to pol γ inhibiting NRTIs: early in the HIV epidemic, in infected patients exposed to high-dose zidovudine therapy; and in uninfected volunteers treated with stavudine. Such measurements have not been performed in contemporary cART treated patients [12], [13].

We have therefore used ^31^P-MRS to determine whether patients on contemporary anti-retroviral therapy have abnormal *in vivo* mitochondrial oxidative function, and whether this correlates with biopsy COX defects.

## Methods

### Participants

Participants were adult HIV-1 infected patients, receiving ambulatory care at one of four specialist clinics (2 hospital-based, 2 community-based setting). Patients with current active hepatitis B or C co-infection were excluded. Participants were unselected with respect to the presence or absence of complications of HIV or anti-retroviral therapy. Patients with known inherited or non-HIV-associated neuromuscular disease were excluded. Demographic data, surrogate markers (CD4 T lymphocyte count, and HIV-1 RNA plasma viral load) and detailed lifetime anti-retroviral treatment history were obtained by case note review.

HIV-uninfected control subjects for ^31^P-MRS studies were age and sex matched to our cases, and we excluded persons with diabetes mellitus or abnormal glucose handling, thyroid disease, previous muscle injury, or diagnosed neuromuscular disease.

Research was approved by the Newcastle and North Tyneside Local Research Ethics Committee (ref. 06/Q0905/137). All subjects gave informed written consent for participation.

### Phosphorus Magnetic Resonance Spectroscopy

MR studies were performed on calf muscle using a 3T Intera Achieva magnet (Philips). ^31^P-MRS measurements were obtained using a calf coil with a voxel within soleus muscle during: a 1 minute baseline resting period; a 3 minute period of calf flexion exercise at 25% of maximal voluntary contractile force; and a 6 minute recovery period [10], [14]. This exercise paradigm was specifically designed to keep metabolism within the aerobic phase. Analysis was performed in jMRUI v3.0 (Java Magnetic Resonance User Interface) using AMARES with appropriate prior knowledge parameters for skeletal muscle [15] and metabolite levels were calculated as previously described [14]. Phosphorylation potential was calculated from the concentration of ATP ([ATP], buffered at 8.2 mM), and the empirically calculated concentrations of adenosine diphosphate and inorganic phosphate ([ADP], [P_i_]), as [ATP]/([ADP][Pi]) [16].

### Skeletal Muscle Biopsies and Mitochondrial (COX) Histochemistry

Percutaneous lower limb skeletal muscle biopsies were performed under local anesthesia and snap-frozen in the liquid phase of isopentane, cooled in liquid nitrogen within 20 minutes of collection. Sequential COX-SDH (cytochrome *c* oxidase/succinate dehydrogenase) histochemistry was performed on 20 µm transverse cryo-sections. COX contains respiratory chain subunits encoded by the mitochondrial genome, and fibers stain brown (positive) in the presence of intact respiratory chain activity. SDH contains subunits encoded entirely by the nuclear genome and thus provides an effective counterstain (blue) as activity will be preserved in the presence of a cellular mtDNA defect. Proportional COX defect was determined by counting ≥500 fibers per biopsy.

### Statistical Comparisons

Student’s paired t-test was used to compare MRS parameters between cases and controls. Correlation coefficients were calculated between COX and MRS data. All analyses were performed in SPSS 19.

## Results

### Patient Characteristics

23 HIV-infected subjects participated; 78% were male. Mean age was 57.6 years, with age range of 45–74 years. Mean duration of diagnosed HIV infection was 11.8 years. Mean current CD4 T lymphocyte count was 551 cells/µl; mean nadir CD4 count was 183 cells/µl. All subjects were currently receiving cART, of whom 96% had a fully suppressed HIV plasma viral load (<40 HIV-1 RNA copies/ml). In addition to their NRTIs, 70% of treated subjects were receiving a non-nucleoside reverse transcriptase inhibitor (NNRTI) and 35% a protease inhibitor (PI). With respect to the pol γ inhibiting NRTIs, 61% of patients had a prior history of zidovudine exposure, and 48% had dideoxynucleoside analog (stavudine, zalcitabine or didanosine) exposure (treatment details of individual patients are shown in **Table 1**).

**Table 1:** Characteristics of HIV-infected subjects.

Subject	Age(yrs)	Gender	Duration of diagnosed HIV infection (mo)	CurrentCD4 count(cells/uL)	CurrentHIV VL(copies/mL)	Nadir CD4 count(cells/uL)	Duration of ART (mo)	Current HAART	Lifetime HAART	Biopsy COX defect (%)
1	55	M	96	503	<40	117	48	TDF FTC ATV/r	TDF FTC ATV/r	0.0%
2	71	M	119	448	<40	UK	119	TDF FTC EFV	ddI AZT 3TC EFV TDF FTC	3.0%
3	74	F	201	825	<40	UK	103	TDF FTC EFV	AZT ddi d4T SQV TDF 3TC EFV	0.4%
4	45	F	71	537	<40	10	70	TDF FTC NVP	AZT 3TC EFV TDF FTC NVP	0.1%
5	55	F	59	406	<40	112	22	TDF FTC AZT DRV/r	TDF FTC LPV/r AZT DRV/r	0.8%
6	63	M	76	783	<40	169	76	ABC 3TC EFV	AZT ABC 3TC EFV	0.0%
7	63	M	215	361	<40	UK	198	ABC 3TC NVP	AZT ddI d4T 3TC ddC IDV NVP ABC	2.2%
8	62	M	43	180	<40	56	42	TDF FTC NVP	TDF FTC NVP	0.2%
9	49	M	193	762	<40	120	193	TDF FTC ATV/r	AZT ddC ddI 3TC d4T SQV NVP IDV NFV ABC TDFLPV/r FTC ATV/r	1.3%
10	48	M	158	872	<40	10	151	TDF ABC NVP	AZT ddI d4T 3TC RTV NVP IDV ddC ABC ATV/r TDF	4.9%
11	60	F	146	666	<40	99	145	ABC 3TC EFV	d4T ABC 3TC EFV	0.2%
12	51	M	141	494	<40	151	140	AZT 3TC NVP	AZT 3TC NVP	1.4%
13	66	M	57	403	<40	287	12	TDF FTC EFV	TDF FTC EFV	11.2%
14	63	F	182	865	<40	300	154	TDF FTC EFV	d4T 3TC NVP NFV EFV AZT TDF FTC	1.2%
15	60	M	101	419	<40	UK	98	TDF FTC NVP	AZT 3TC EFV NVP TDF FTC	2.4%
16	61	M	262	422	<40	UK	160	ABC NVP LPV/r	SQV AZT ddC 3TC d4T ddI IDV ABC NVP NFV LPV/r	0.8%
17	54	M	66	603	<40	244	25	TDF FTC DRV/r	TDF FTC EFV DRV/r	3.4%
18	51	M	237	559	<40	327	165	TDF FTC EFV	AZT ddI RTV NFV TDF FTC EFV	1.5%
19	62	M	143	329	<40	163	55	TDF FTC DRV/r	AZT 3TC NVP FOS-APV RTV TDF FTC DRV/r	1.2%
20	50	F	120	1358	<40	541	0	nil	nil	0.0%
21	53	M	UK	804	<40	301	48	TDF FTC EFV	TDF FTC EFV	NA
22	56	M	240	401	97	150	224	TDF FTC ETR DRV/r	AZT ddC SQV 3TC IDV d4T NVP ddI ABC LPV/r TDFATV/r FOS-APV/r DRV/r MVC FTC	2.2%
23	45	M	165	592	<40	305	146	RAL ABC ATV/r	d4T 3TC NVP ddI IDV ABC ATV/r RAL	9.8%
24	57	M	145	435	<40	379	21	TDF FTC EFV	TDF FTC EFV	0.4%

VL, plasma HIV-1 RNA viral load; (c)ART, (combination) anti-retroviral therapy; AZT, zidovudine; d4T, stavudine; ddI, didanosine; ddC, zalcitabine; 3TC, lamivudine; ABC, abacavir; TDF, tenofovir; FTC, emtricitabine; EFV, efavirenz; NVP, nevirapine; ATV, atazanavir; DRV, darunavir; LPV, lopinavir; SQV, saquinavir; NFV, nelfinavir; IDV, indinavir; FOS-APV, fosamprenavir; RTV, ritonavir at therapeutic dose;/r, ritonavir at pharmacokinetic boosting dose; MVC, maraviroc; RAL, raltegravir; UK, unknown; NA, not available; COX, cytochrome c oxidase. COX data (but not MRS data) from 5 subjects has been previously described [8].

### Measures of Muscle ATP and Acid Metabolism by ^31^P-MRS

**Figure 1:**
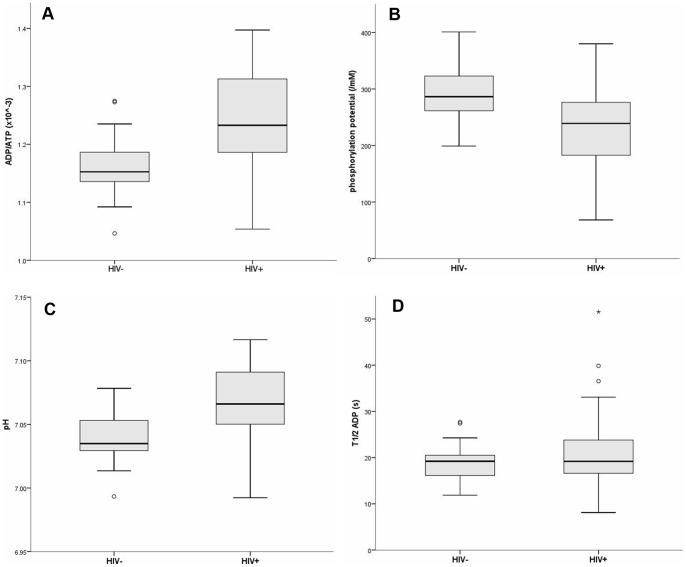
Phosphorus magnetic resonance spectroscopy. Resting state metabolic parameters differed significantly between HIV-infected subjects (HIV+) and HIV-uninfected controls (HIV−): ADP/ATP (adenosine diphosphate/ATP) ratio (**a**), phosphorylation potential (**b**), and pH (**c**) (n = 23 each; ADP/ATP, p = 0.001; phosphorylation potential, p = 0.003; pH, p = 0.002). In contrast, the rate of ATP re-synthesis (estimated as τ_½_ ADP) following exertion was not significantly impaired in HIV-infected subjects compared with controls (p = 0.09) (**d**).

In the resting state, baseline ATP metabolites and pH values were significantly higher in cART-treated HIV-infected subjects compared to age and gender-matched controls (mean ±SD): ADP/ATP ratio, HIV-infected 1.24±0.08×10^−3^, HIV-uninfected 1.16±0.05×10^−3^, p = 0.001; phosphocreatine/ATP (PCr/ATP) ratio, HIV-infected 5.04±1.89, HIV-uninfected 3.75±0.26, p = 0.004; pH, HIV-infected 7.07±0.03, HIV-uninfected 7.04±0.02, p = 0.002. Correspondingly, calculated basal phosphorylation potential was significantly lower in HIV-infected subjects compared with controls: HIV-infected 227±86 mM^−1^, HIV-uninfected 292±53 mM^−1^, p = 0.003 (**Figure 1a–c**). (Further details of calculated ^31^P-MRS parameters are shown in the **Table S1.**).

In terms of dynamic oxidative function, mean post-exercise ATP metabolite recovery rates did not differ significantly between HIV-infected subjects and controls. For example, τ_½_ ADP: HIV-infected 22.1±9.9 s, HIV-uninfected 18.8±4.4 s, p = 0.09 (**Figure 1d**). None of the clinical variables (age, duration of diagnosed HIV infection, CD4 T lymphocyte count, or anti-retroviral treatment history) correlated significantly with any of the baseline or post-exercise ^31^P-MRS parameters in HIV-infected subjects.

### Mitochondrial (COX) Histochemistry and Correlation with ATP Metabolism

**Figure 2:**
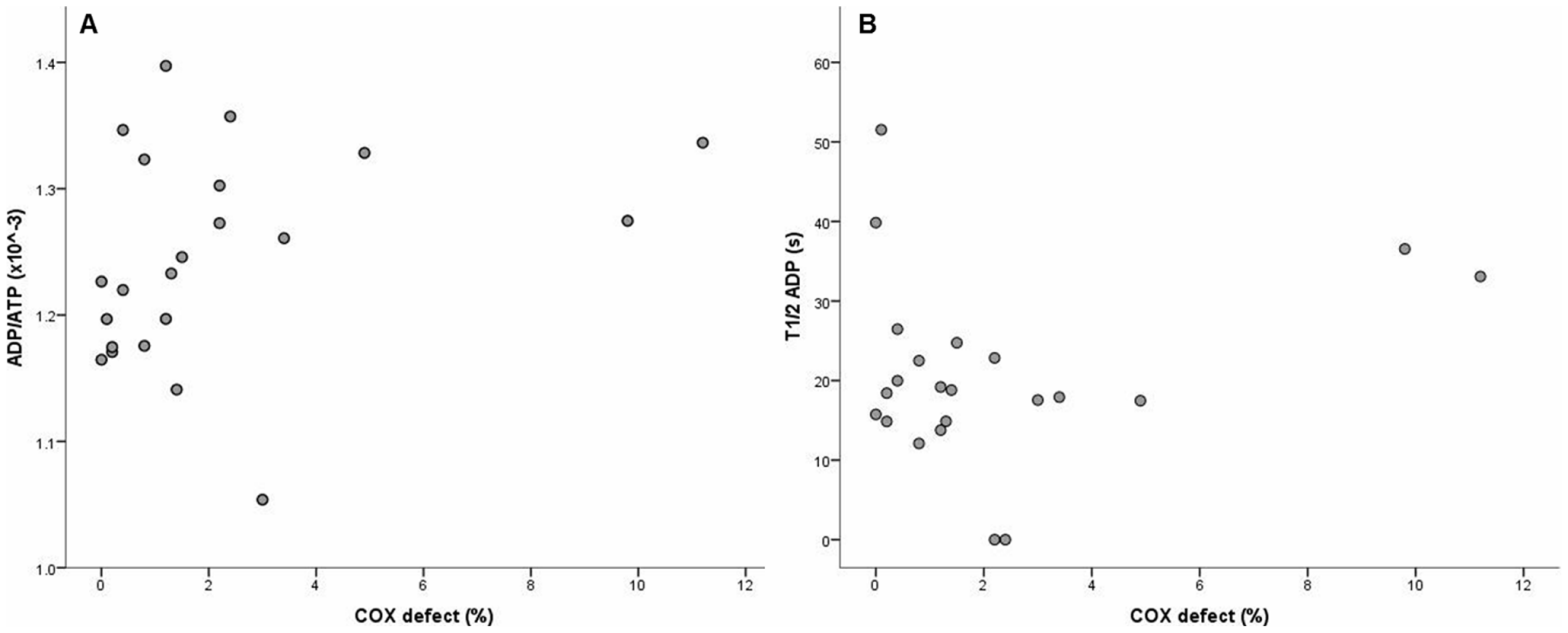
Relationship of phosphorus magnetic resonance spectroscopy and muscle histochemistry. Resting state ADP/ATP ratio showed moderate correlation with the percentage frequency of COX deficient muscle fibers in treated HIV-infected subjects (Kendall’s τ = 0.34, p = 0.034) (**a**), whereas the rate of ATP re-synthesis following exertion (estimated as τ_½_ ADP) did not (Kendall’s τ = 0.04) (**b**).

A wide range of COX defects were observed across the subject group (0 to >10% of muscle fibers affected per biopsy). Interestingly, we observed significant COX defects both in patients with prior exposure to pol γ inhibiting NRTIs, and in some patients without such exposure (COX defects for individual patients are shown in **Table 1**). Resting state ADP/ATP ratio showed a moderate correlation with biopsy proportional COX defect (Kendall’s τ = 0.34, p = 0.034) (**Figure 2a**). There was no correlation between dynamic ATP metabolism, for example as estimated by τ_½_ ADP, and biopsy COX defect (**Figure 2b**).

## Discussion

In our study, most anti-retroviral treated HIV-infected subjects demonstrated dynamic *in vivo* tissue mitochondrial function comparable with uninfected control subjects, whereas it is generally impaired in inherited mitochondrial disorders. Our subject group included patients with very long durations of HIV infection and extensive anti-retroviral drug treatment histories, including past exposure to pol γ inhibiting NRTIs. Interestingly, in the present study we observed COX defects both in subjects with prior exposure to pol γ inhibiting NRTIs, and in some subjects without such exposure. This observation contrasts with our previous work in younger HIV-infected patients (all aged ≤50 years), where COX defects appeared to be attributable almost entirely to exposure to pol γ inhibiting NRTIs [8]. In the present study, the heterogeneous COX defects are most likely to reflect the significantly older subject age range (45–74 years). In this age group it is expected to see some COX defects due to normal aging [17], although it is also possible that there are other unmeasured HIV or treatment-associated factors driving COX defects in some of these patients. As with NRTI-associated COX defects, these COX deficient fibers also contain high levels of individual somatic mtDNA mutations [18]. What is therefore the likely explanation of our finding of largely normal *in vivo* muscle mitochondrial function? COX-deficient fibers contain high levels of mutant mtDNA, whereas COX positive fibers contain almost exclusively wild-type mtDNA. Therefore function at the whole tissue level is presumably compensated by the larger number of fibers with normal COX function. In contrast, in the historical context when patients were actively treated with a pol γ inhibiting NRTI, the molecular defect was one of mtDNA depletion, and the impairment of oxidative function would be expected to affect all fibers [13]. We therefore conclude that although somatic mtDNA mutations and associated cellular COX defects are frequently present in contemporary cART treated patients, there is some ability to compensate for *in vivo* exertional oxidative function in the muscle as a whole.

The decreased basal phosphorylation potential, as we have observed in our patients, implies an increased rate of ATP synthesis at rest [19]. Cytosolic ATP concentration is tightly buffered, and results from the balance of the ATP hydrolysis required for the maintenance of cellular integrity and the synthesis of ATP from oxidative phosphorylation [16]. The rate of ATP synthesis is strongly dependent on the phosphorylation potential in resting muscle, with a decreased phosphorylation potential, as we have observed in our patients, implying an increased rate of ATP synthesis [19]. This notion suggests that there is a requirement for an increased basal rate of intracellular ATP hydrolysis in HIV-infected subjects, and an increased basal rate of ATP synthesis is therefore required to maintain ATP homeostasis. Although this might imply increased basal energy expenditure in these patients compared with healthy subjects, given that dynamic *in vivo* mitochondrial function is unimpaired, the physiological significance of this observation remains uncertain. Further work should therefore examine correlates of this finding, such as fatigue [20].

In conclusion, in a cohort of predominantly older HIV-infected patients with longstanding cART, we observed frequent histochemical COX defects both in patients with and without prior exposure to pol γ inhibiting NRTIs. It is, however, broadly reassuring that *in vivo* whole tissue mitochondrial function in most contemporary anti-retroviral treated patients appears to be largely maintained, despite the presence of this frequent mitochondrial damage within individual cells.

## Supporting Information

Table S1 **Phosphorus magnetic resonance data.** Calculated ^31^P-MRS parameters, in resting state and during recovery from sub-maximal exercise.
(DOCX)
