# Supplementary material for: In Vivo Mitochondrial Function in HIV-Infected Persons Treated with Contemporary Anti-Retroviral Therapy: A Magnetic Resonance Spectroscopy Study
Source: PLoS One. 2014 Jan 7;9(1):e84678. doi: 10.1371/journal.pone.0084678 (PMC3883680; doi:10.1371/journal.pone.0084678)
Supplement: Table S1 — Phosphorus magnetic resonance data. Calculated 31P-MRS parameters, in resting state and during recovery from sub-maximal exercise. (DOCX) [file pone.0084678.s001.docx]

**Online Supplementary Material**

***PLOS ONE* – Research Articles**

**Title:** *In vivo* mitochondrial function in HIV-infected persons treated with contemporary anti-retroviral therapy: a magnetic resonance spectroscopy study.

**Authors:** Brendan AI Payne *et al*.

**Supplementary Table S1. Phosphorus magnetic resonance data.**

|  | **HIV (n = 23)** | | **Control (n = 23)** | |  |
| --- | --- | --- | --- | --- | --- |
|  | **mean** | **SD** | **mean** | **SD** | **p-value** |
| **Basal (Resting state)** |  |  |  |  |  |
| P_i_/ATP | 0.52 | 0.27 | 0.37 | 0.06 | **0.010** |
| PCr/ATP | 5.04 | 1.89 | 3.75 | 0.26 | **0.004** |
| ADP/ATP (x10^-3^) | 1.24 | 0.08 | 1.16 | 0.05 | **0.001** |
| Phosphorylation potential (mM^-1^) | 227 | 86 | 292 | 53 | **0.003** |
| pH | 7.07 | 0.03 | 7.04 | 0.02 | **0.002** |
|  |  |  |  |  |  |
| **Post-exercise (Recovery)** |  |  |  |  |  |
| Initial PCr resynthesis rate (mM/min) | 15.6 | 14.8 | 12.2 | 4.9 | 0.22 |
| τ½ PCr (s) | 30.2 | 13.6 | 27.1 | 8.3 | 0.31 |
| τ½ ADP (s) | 22.1 | 9.9 | 18.8 | 4.4 | 0.09 |
| Q_max_[ATP] (mM/min) | 27.5 | 19.2 | 23.3 | 10.2 | 0.28 |
| Minimum pH | 6.98 | 0.14 | 7.00 | 0.03 | 0.48 |
| Initial proton efflux (mM/min) | 2.37 | 2.10 | 1.69 | 1.70 | 0.10 |

(P_i_, inorganic phosphate; PCr, phosphocreatine; ADP, adenosine diphosphate; ATP, adenosine triphosphate.)
